# Environmentally Friendly Bleaching Process of the Cellulose Fibres Materials Using Ozone and Hydrogen Peroxide in the Gas Phase

**DOI:** 10.3390/ma17061355

**Published:** 2024-03-15

**Authors:** Anetta Walawska, Magdalena Olak-Kucharczyk, Anna Kaczmarek, Marcin H. Kudzin

**Affiliations:** Łukasiewicz Research Network—Łódź Institute of Technology, 19/27 Marii Sklodowskiej-Curie Str., 90-570 Łódź, Poland

**Keywords:** bleaching, cellulose, cotton, hydrogen peroxide, hydrogen peroxide vapours, ozone, decontamination, fibre materials, polymer functionalization

## Abstract

The paper presents the new eco-friendly method of bleaching process of the cellulose fibre materials. Cellulose materials were bleached using hydrogen peroxide (both in aqueous solution, vapours, ozone and by the combined action of gaseous hydrogen peroxide and ozone. The method using hydrogen peroxide in aqueous solution presents the standard procedure and was used as the comparison technique. The bleaching processes using gaseous oxidants were carried out in a prototype device for dry, low-temperature treatment of fibrous materials with the use of oxidising agents in the gas phase. The influence of the innovative gas-phase bleaching method on the cotton samples’ properties was analysed by Scanning Electron Microscopy (SEM), evaluation of the colour and whiteness, assessment of the polymerisation degree (DP), analysis of the mechanical properties and sorption capacity as well as microbiological assessment against colonies of Gram-positive (*Staphylococcus aureus*) and Gram-negative (*Escherichia coli*) bacteria. The comparison of the obtained results led to the conclusion that the bleaching processes using gas-phase agents—vaporised hydrogen peroxide, ozone or their combination—are non-invasive. The applied bleaching processes resulted in a slightly lower whiteness parameters than standard bath bleaching. After the bleaching processes with ozone and vaporised hydrogen peroxide separately, the decrease in the DP and tensile strength was similar to that observed after the bleaching with aqueous H_2_O_2_. When both processes were used together, a higher reduction in DP and tensile strength was noticed. Both oxidising agents showed a strong biocidal effect against bacteria. Gas-phase bleaching procedures, due to the lower temperature (35 °C vs. 98 °C) and minimal water consumption, have economic and environmental advantages, which allows their use in semi-industrial applications. It has been shown that the treatment of cotton fabrics using ozone and hydrogen peroxide in the gas phase allows to simultaneously obtain the bleaching and disinfection effect.

## 1. Introduction

Cotton fibre is the second most widely used fibre in the textile industry with a global production of 26.2 million tons and a 24.4% textile fibre market share in 2020 [1,2]. These fibres are a basic material widely used in the clothing and apparel industry [3], for medical and hygienic purposes [4,5], and also for environmental applications [6]. The major component of cotton fibres is cellulose—a natural polysaccharide made of glucose molecules connected by β-1,4-glycosidic bonds [7,8,9]. Nearly all cotton fibres produced in the world are white; however, a few types of cotton occur in colours ranging from white to various shades of green, cream and/or brown [10,11].

Raw cotton contains a number of non-cellulosic materials that are generally considered to be surface-related and may therefore affect fibre quality. The main constituents of the cotton causing its colour are lignin, flavonoids and wax. These compounds together with the structure of cellulose are presented in Table 1.

A removal of colorizing additivities from cotton is carried out during the bleaching processes, which are to a great extent based on oxidative treatments. Nowadays, textile manufacturing is a multistage process in which the bleaching process is inextricably linked with textile wastewater treatment. Due to the size of the global textile market and its environmental impact, the development of effective, economical, and easy-to-handle alternative treatment technologies for textile wastewater is of significant interest [21].

The large cotton production causes significant amounts of waste. Every year, 11.6 million tons of cotton waste are generated worldwide [1,22]. One of the priority directions in the field of wet processing of textiles is directed towards shortening and simplification of the processing sequence influencing the water consumption and impact on the natural environment [23,24].

Therefore, new procedures enabling “transfer from wet to dry environment” are of great importance. Such procedures include a gas-phase bleaching, with potential application of gaseous oxidants such as oxygen, ozone, chlorine, chlorine dioxide and/or hydrogen peroxide [25,26,27,28].

However, chlorine components, such as chlorine and hypochlorite, present limited use due to their reactivity [29,30,31], toxicity [32] and harmful environmental impact [33]. Chlorine dioxide gas has an excellent ability for sterilization and deodorization and is harmless to humans [34]. However, it is unstable and explosive (in high concentration) [35], which limits its potential application. Therefore, ozone and hydrogen peroxide are components of choice. These reagents react with alkene and phenolic fragments (see lignins, flavones and pigments in Table 1) by free-radical addition to double bond, converting them to temporary carbonyl and finally carboxyl compounds, easily removable during the washing process [36,37,38,39,40]. Hydrogen peroxide is an unstable compound with a boiling temperature of 150 °C (lower under decreased pressure) [41]. It forms stable hydrosols/aerosols (aHP) [42]. The physical chemistry of the vaporisation of hydrogen peroxide has been reviewed by Hultman et al. [43]. A review of the medical application of various physical forms of hydrogen peroxide, including aerosolized hydrogen peroxide (aHP) and vaporised hydrogen peroxide (VHP), has been published recently [44]. Vaporised hydrogen peroxide is produced by the vaporisation of a liquid, acidic hydrogen peroxide at 120 °C. As a result, a mixture of VHP and water vapour is obtained.

Pironti [45] recently used hydrogen peroxide vapour as a disinfectant in the vapour phase to treat fabrics and textiles (cotton, linen, silk, wool, polyester, viscose). Similarly, Wawrzyk [46] applied H_2_O_2_ for disinfecting historical cotton textiles from the Auschwitz-Birkenau State Museum in Oświęcim, Poland. There are few reports in the scientific literature about the use of vaporised hydrogen peroxide for the bleaching of textile products. In recent years, VHP has been used by the authors of this paper for bleaching and disinfection of cotton textile products [47]. This innovative, waste-free and low-temperature process allows to obtain bleached textile products characterized by an acceptable whiteness and microbiological purity, devoid of the following microorganisms: bacteria—*Staphylococcus aureus*, spores *Bacillus subtilis* and *Bacillus atrophaeus*, as well as fungi—*Chaetomium globusom* and *Aspergillus niger*. This process can be an alternative to the conventional water- and energy-consuming bleaching process using aqueous hydrogen peroxide for products with special applications, such as medical (gauze, cotton wool, bandages) and hygienic—protective masks for the human respiratory system used as a prevention of bacterial and/or viral disease transmission [47].

Ozone has strong oxidising properties, which is why it has a harmful effect on various forms of life. Ozone destroys bacteria, viruses and fungi, due to the destructive effect on the protective cell walls of these microorganisms. For this reason, it is widely used for the decontamination purposes in several industries [48], e.g., for water [49,50] and wastewater [51,52,53,54,55] treatment, including textile wastewater [56,57,58], in the food industry [59,60,61,62], in the paper industry—for bleaching cellulose pulp [63,64], and in the pharmaceutical and medical sectors [48,65]. The emergence of the COVID-19 pandemic has led to the recent developments in the implementation of various ozone-based technologies for the disinfection of surfaces, materials and the indoor environment [48,66,67,68,69,70]. There are also reports regarding the use of ozone for the bleaching of cotton fibres [71], jute, silk, angora [72], mohair [73] and soybean fabrics [74,75,76,77]. In the above studies, ozone gas was introduced into the bleaching (water) bath. As well as that, there are studies regarding the use of ozone in the gas phase for the oxidative treatment of cotton fibre materials [78,79], including the processing of jeans [80].

The innovative solution proposed in this paper describes the combined use of two oxidising agents in the gas phase: vaporised hydrogen peroxide (VHP) and ozone, for advanced/deep oxidation of organic impurities in cellulose fibres. Both agents are active at low temperatures (ambient temperature) and do not produce products harmful to the environment, which makes the process not only ecological but also economical. The current study investigates the possible synergistic effect of the treatment of fibrous cellulosic materials using a combination of VHP and ozone on the physicochemical properties of the fabrics in comparison to conventional bleaching processes.

## 2. Materials and Methods 

### 2.1. Materials

- Unbleached 100% plain woven cotton fabric with a weight of 170 g/m^2^. The amount of weft yarn in the longitudinal direction per unit length was 20 yarns/cm, and the amount of warp yarns in the transverse direction per unit length was 16 yarns/cm. Before testing, cotton fabric samples were subjected to pre-treatment with an anionic wetting–washing agent—Periwet WLV (Dr. Petry GmbH, Reutlingen, Germany)—2 g/L, 98 °C, 30 min, using laboratory dyeing machine RED KROME(Ugolini S.R.L., Schio, Italy)
- Hydrogen peroxide solution 30% (CAS No.: 7722-84-1) was purchased from Millipore Sigma (St. Louis, MO, USA).
- Bacterial strains: *Staphylococcus aureus* (ATCC 6538, Gram-positive bacteria) and *Escherichia coli* (ATCC 25922, Gram-negative bacteria) were purchased from Microbiologics (St. Cloud, MN, USA).

### 2.2. Bleaching Process of Cellulose Fibrous Materials

Cellulose-fibre materials made from cotton (COT) were subjected to the bleaching process using vaporised hydrogen peroxide (H_2_O_2(g)_; VHP) and ozone (O_3(g)_). Table 2 presents abbreviations of the cotton samples used for the investigations. Since the parts of these abbreviations contain chemical formulas, we used COT for cotton instead of CO/Co (CO and/or Co can be confused with carbon oxide or cobalt). The resulting cotton samples and the applied conditions of the bleaching procedures are listed in Table 2.

**Figure 1:**
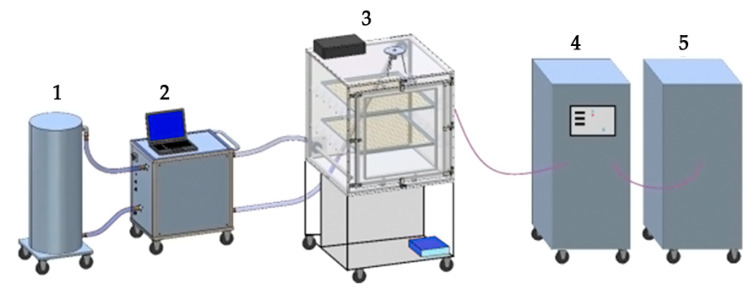
The prototype device for dry, low-temperature treatment of fibrous materials with the use of oxidising agents in the gas phase, i.e., hydrogen peroxide and/or ozone (1—absorption column with a filter; 2—Central unit with a VHP generator; 3—working chamber; 4—ozone generator; 5—oxygen concentrator).

The processes were carried out in a prototype device for dry, low-temperature treatment of fibrous materials with the use of oxidising agents in the gas phase. The apparatus is schematically presented in Figure 1 and consists of the following elements:

- Absorption column with a filter (1);
- Central unit in which a generator of vaporised (gaseous) hydrogen peroxide is placed, allowing to set the parameters of the process and to control the readings from sensors placed in the chamber (2);
- Hermetic sealed working chamber having a volume of approximately 1 m^3^, made of transparent plastic resistant to hydrogen peroxide (acrylic glass), equipped with a fan and a set of sensors enabling to control relative humidity, temperature, concentration of oxidising agent, pressure (3);
- Ozone generator, allowing to provide the ozone concentration in the chamber up to approximately 60 g/m^3^ (4);
- Oxygen concentrator (5).

#### 2.2.1. Bleaching Process of Cellulose Materials Using Hydrogen Peroxide in the Gas Phase (H_2_O_2(g)_, VHP)

The hydrogen peroxide concentration inside the working chamber was equal to 800 ppm (0.24 Mol/L), the temperature was set to 35 °C, while the hydrogen peroxide treatment time was equal to 1 or 4 h, and the aeration time was 24 h (sample COT-H_2_O_2(g)_). The applied bleaching process consists of the following steps:

- Dehumidification—the drying of the working chamber in order to lower the relative humidity to 40 ± 3% in order to allow the higher concentration of the active vapours;
- Saturation—dosing of hydrogen peroxide in order to achieve the proper concentration of hydrogen peroxide vapours (H_2_O_2(g)_, VHP) inside the working chamber (800 ppm; 0.24 Mol/L);
- Bleaching with a simultaneous decontamination—in this phase, the concentration of the hydrogen peroxide in the form of dry gas (below the condensation point) is maintained at the previously defined level for a certain period of time; the system constantly supplies and replenishes hydrogen peroxide in the working chamber, while the installed fans ensure the even distribution of air, VPH and water vapour within the entire volume of the chamber;
- Aeration—exchange of air inside the working chamber or the decomposition of the active substance; in this step, the hydrogen peroxide supply to the working chamber is stopped, and hydrogen peroxide mixed with water vapour is exhausted from the working chamber (in a closed cycle) until a safe concentration is reached.

#### 2.2.2. Bleaching Process of Cellulose Materials Using Ozone

The ozone bleaching was carried out under the following conditions: the ozone concentration within the working chamber was equal to 1000–15,000 ppm (0.021–0.31 Mol/L), the temperature was set to 35 °C, while the time was equal to 0.25 or 1 h (sample COT-O_3(g)_). Ozone was produced at the moment of use by electrical discharge that was provided by an ozone generator (Figure 1).

#### 2.2.3. Bleaching Process of Cellulose Materials Using Combination of VHP and Ozone

The innovative bleaching procedure proposed in this paper consisted of a sequential bleaching using VHP and ozone in a gas phase. Firstly, the samples were treated using hydrogen peroxide in the gas phase. The hydrogen peroxide concentration was equal to 800 ppm (0.24 Mol/L), the temperature was 35 °C, while the hydrogen peroxide treatment time was equal to 1 h. Next, the samples were treated with gaseous ozone. The applied concentration of ozone was equal to either 1000 or 10,000 ppm (0.021 or 0.21 Mol/L), the temperature was set to 35 °C, while the time was equal to 1 h.

#### 2.2.4. Conventional Bleaching Process

For a comparison, samples made from cotton fabric were also subjected to a conventional bleaching process in the laboratory using an Ugolini dyeing machine, model: Redcrome/RED/P (Schio, Italy), in a bath containing 4 g/L of 30% H_2_O_2(as)_ and auxiliary agents (sequestering agent Contavan GAL (CHT Germany GmbH, Dußlingen, Germany)—organic chelate former based on hydroxycarboxylic acids—1.0 g/L) and wetting/washing agent (Periwet WLV (Dr. Petry GmbH, Reutlingen, Germany)—1.5 g/L) at 98 °C for 60 min (sample COT-H_2_O_2(as)_). The liquor ratio was 1:10. 

### 2.3. Analytical Methods 

#### 2.3.1. Scanning Electron Microscopy (SEM)

The morphology of the investigated samples was examined using the Tescan Vega 3 scanning electron microscope (Brno, Czech Republic). The samples were observed using a secondary electron imaging mode, under high vacuum and with the accelerating voltage equal to 20 kV. Prior to the analysis, the surface of all samples was covered with a thin layer of gold using a Quorum Technologies Ltd. vacuum sprayer (Lewes, UK).

#### 2.3.2. Colour and Whiteness Measurement

##### Evaluation of the Whiteness

The whiteness of the cotton samples after the applied bleaching processes was assessed by the evaluation of the CIE whiteness index (WI_CIE_) according to the EN ISO 105-J02:2002 standard [81]. The measurements were performed using the Datacolor 650 spectrophotometer (Datacolor Int., Luzern, Schweiz) with the d/8 geometry, for the 10° observer and by the D65 light. The obtained data were analysed by means of the Datacolor Tools (version 1.3.1) software. The colorimetric coordinates (X, Y, Z) and the chromaticity coordinates (x, y) were determined for each sample. Next, the whiteness index was calculated using the CIE formula (Equation (1)):

WI_CIE_ = Y + 800(x_0_ − x) + 1700(y_0_ − y) (1)

where x_0_ and y_0_ are the coordinates of the achromatic point for the given illuminate (for the D65 light and 10° observer, these values are equal to 0.3138 and 0.3310, respectively). The above WI_CIE_ index is limited to the value 40 < WI_CIE_ < 5Y − 280. The investigations on the predictive modelling of the cotton fabric’s whiteness index, in COT-NaOH-H_2_O_2(as)_ for various reagent concentrations and bleaching conditions, were carried out recently [82].

##### Yellowness Index 

The degree of yellowness—E313 Yellowness Index (YI)—of the textile samples treated with vaporised hydrogen peroxide and ozone was calculated according to the ASTM Method E313, Standard Practice for Calculating Yellowness and Whiteness Indices from Instrumentally Measured Colour Coordinates” (2020) [83], and according to Equation (2):

YI = (100(CxX − CzZ))/Y(2)

where X, Y and Z are the CIE colour coordinates, while the value of the Cx and Cz factors depends on the illuminate and the observer. For the D65 illuminate and the 10° observer, the values of these coefficients are, respectively: 1.3013 and 1.1498. 

##### Evaluation of the Colour

The colour of the cellulose samples after the applied bleaching processes was assessed according to the EN ISO 105-J02:2002 standard using the CIELAB technique [46]. For that purpose, the Datacolor 650 spectrophotometer (Datacolor Int., Luzern, Schweiz) with the d/8 geometry equipped with the Datacolor TOOLS, Version: 1.3.1, Build: 514 software was used. The CIE colour coordinates (L*, a*, b*, C* and h) were determined for the D65 light and 10° observer using the wide measuring window (LAV) with a diameter equal to 30 mm. The difference in the colour (DE) in comparison to the specific standard, i.e., control sample not subjected to the bleaching process, was determined according to EN ISO 105-J03:2009 [84].

#### 2.3.3. Determination of the Polymerisation Degree

The degree of the polymerisation of the cellulose samples was determined according to EN ISO 5351:2010 [85]. The procedure is based on the measurement of the viscosity ratio of the sample solution in bis(ethylenediamine)copper(II) hydroxide solution (Cupri-EthyleneDiamine—CED) by measuring the efflux time of the solution. For that purpose, 0.2500 g of moisture-free cellulose (cotton fabric sample) was dissolved in 50 mL of solution consisting of 25 mL of CED and 25 mL of deionized water. The cellulose solution was thermostated at 25 °C and introduced into a viscometer with a glass capillary. Then, the flow time of the liquid through the capillary was measured. On this basis, the relative viscosity ŋ of the tested solution was calculated, according to the following formula (Equation (3)):

ŋ = h∙t(3)

where h is the previously determined viscometer constant, and t is the flow time of the tested solution through the capillary.

Then, the concentration of the solution C was calculated according to Equation (4):

C = (2∙m∙s/100)∙100(4)

where m is the mass of the sample in grams, and s is the mass dryness in %.

Knowing the relative viscosity, the value of the [ŋ]·C was determined and then, knowing the concentration of the solution, the intrinsic viscosity [ŋ] of the solution was calculated. Finally, the degree of the polymerisation was calculated using the Immergut, Shurz and Mark formula [86], which describes the relationship between the degree of the polymerisation of linear chain compounds and the intrinsic viscosity of the solution (Equation (5)):

DP^a^ = K·[ŋ] (5)

where DP is the polymerisation degree, [ŋ] is the intrinsic viscosity in g/cm^3^, and a and K are the constants dependent on the type of the solvent (a = 0.905 and K = 0.75 for 1 mol/L solution of ethylenediamine copper (II) hydroxide).

#### 2.3.4. Assessment of the Mechanical Properties

Tensile strength and elongation at break were assessed using a Hounsfield H5KS (Tinius Ltd., England, UK) tensile testing machine according to EN ISO 13934-1:2013 [87]. The samples with a width of 50 mm and length of 200 mm were stretched at a constant elongation rate equal to 100 m/min until the breakage. The maximum force and elongation at maximum force of the examined cellulose materials were registered. Each measurement was pentaplicated (both in warp and weft directions). 

#### 2.3.5. Determination of the pH of an Aqueous Extract

For the samples made of woven cotton fabric, the pH of the aqueous extract was determined according to EN ISO 3071:2020 [88]. For that purpose, the laboratory test samples of unmodified cotton and of cotton bleached using different procedures were cut into pieces having approximately a 5 mm side (or into pieces of the size allowing to wet rapidly). From each sample, two specimens were taken—2.00 ± 0.05 g each. Then, each specimen was placed in a stoppered flask containing 100 mL of the extracting solution and was shaken mechanically for 2 h ± 5 min. Distilled water with a pH of 5.8 and a temperature of 24.1 °C was used as a solvent.

#### 2.3.6. Measurement of the Sorption Capacity

Water sorption capability of the examined cotton samples in superficial contact with a liquid was tested using the SORP-3 instrument (KONTECH, Lodz, Poland). The method is based on the measurement of the quantity of water absorbed by a unit surface of the sample as a function of time [89]. The following parameters were determined: the maximum sorption—S_max_ (expressed as the µL of the absorbed liquid per cm^2^ of the sample), the total sorption time t_max_ (expressed in s), the maximum sorption velocity V_max_ (µL/cm^2^s) and sorption velocity V_30–70_ (µL/cm^2^s). Tests were carried out using distilled water and L/S 14 tubes. The pressure exerted on the samples was equal to 0.5 kPa.

#### 2.3.7. Microbiological Assessment of the Decontamination Efficiency

The decontamination efficiency of the bleaching procedures was assessed using the Gram (+) *Staphylococcus aureus* (strain ATCC 6538, Microbiologics, St. Cloud, MN, USA) and Gram (-) *Escherichia coli* (strain ATCC 11229, Microbiologics, St. Cloud, MN, USA) bacteria. The following concentrations of inoculum were used: *E. coli*—CFU/mL = 6.3 × 10^6^, *S. aureus*—CFU/mL = 0.9 × 10^7^. The bacteria were deposited on the surface of the samples (1 cm^2^) using 0.1 mL of suspension per sample. Afterwards, the contaminated samples were subjected to the bleaching process using ozone (10,000 ppm, 0.21 M; 0.33 h) or vaporised hydrogen peroxide (800 ppm; 0.024 M, 0.33 h). Next, the samples were placed into the proper broth (tryptone soya broth, Oxoid, UK) and incubated for 72 h at 37 °C in order to provide the adequate conditions for bacteria growth. The samples made from woven cotton fabric contaminated with the selected microorganisms and not subjected to the bleaching process were used as control samples. The samples were considered sterile if there was no bacteria growth in the broth after the incubation time.

## 3. Results and Discussion

### 3.1. Bleaching

**Figure 2:**
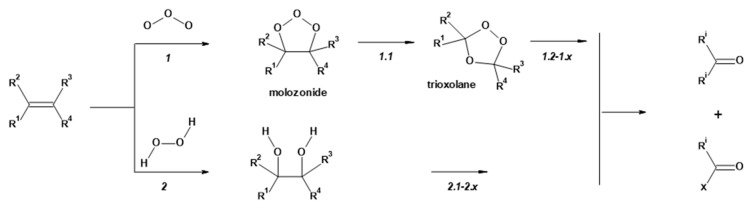
The simplified reaction scheme of alkenes (structural constituents of lignin, flavonoids and cotton fibre wax (Table 1)) with ozone (reaction 1, and subsequent reactions 1.1 to 1.x) and hydroxyperoxide (reaction 2, and subsequent reactions 2.1 to 2.x).

The basic principle of the applied bleaching methods results from reactivity differences of alkenes/polyhydroxyphenols (constituents of lignins, flavonoids and waxes) and cellulose (major constituent of cotton) (Table 1) during the bleaching process. Thus, the reactions of alkenes (polyhydroxyphenols) with ozone [90] or hydrogen peroxide [36,91] occur quickly and quantitatively, whereas cellulose undergo only very slow changes [36] (Figure 2).

As a result of the ozone/hydrogen peroxide treatment, the cotton structural constituents—lignins, flavonoids and waxes—covert into low-molecular, soluble-in-water compounds (carbonyls and carboxylic acids), which in turn are removed during the subsequent washing.

### 3.2. Scanning Electron Microscopy (SEM) 

**Figure 3:**
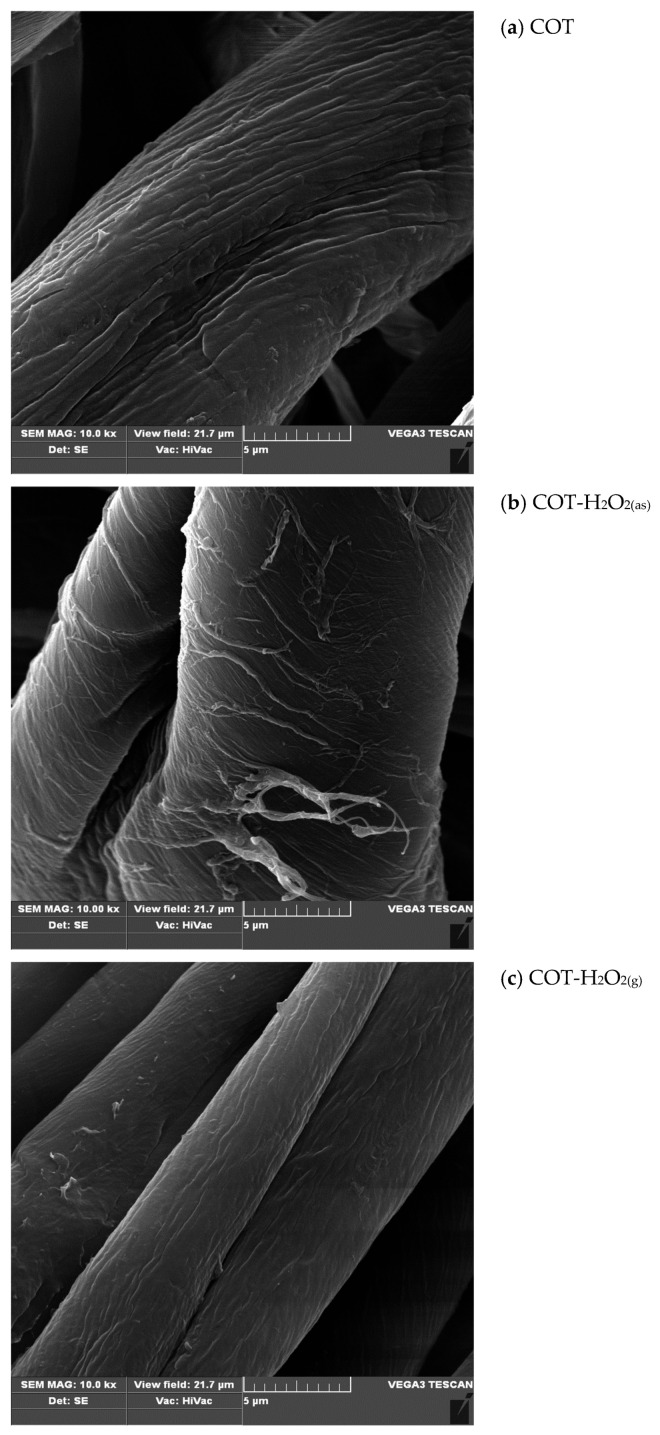

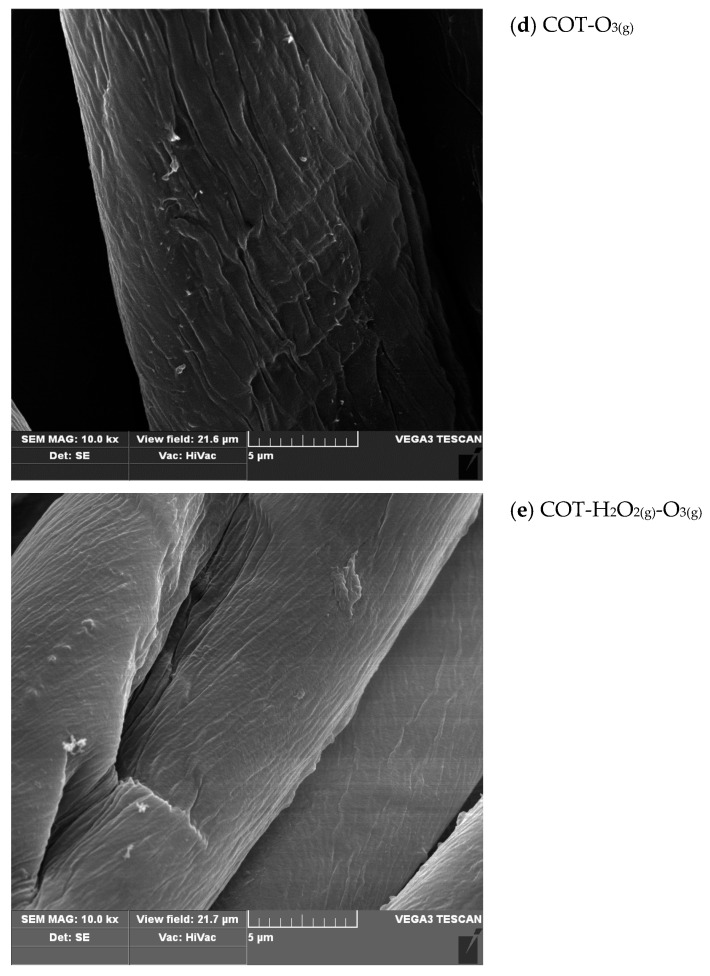
SEM images of the investigated samples, namely the raw cotton ((**a**); COT) and the cotton samples bleached in the hydrogen peroxide aqueous solution ((**b**), COT-H_2_O_2(as)_) and in the gaseous oxidant environments ((**c**), COT-H_2_O_2(g)_; (**d**) COT-O_3(g)_; and (**e**) COT-H_2_O_2(g)_-O_3(g)_). The magnifications of all SEM images (**a**–**e**) = 10,000×; scale bar = 5 μm).

SEM micrographs of the investigated cotton samples are presented in Figure 3. These include images of cotton samples subjected to the bleaching in the hydrogen peroxide aqueous solution (COT-H_2_O_2(as)_), and the images of cotton samples bleached in gaseous oxidant environments (oxidants: H_2_O_2(g)_; O_3(g)_; H_2_O_2(g)_ + O_3(g)_; samples: COT-H_2_O_2(g)_; COT-O_3(g)_; COT-H_2_O_2(g)_-O_3(g)_, correspondingly), using the raw cotton sample as the SEM image control.

It may be observed that all of the applied bleaching treatments resulted in a slight change in the surface morphology of the fibres. This is due to the removal of lignins, flavons and wax deposits from the fibres’ surface. As a result, the cracks on the surface of the cellulose fibres were observed. These changes are most noticeable for fibres subjected to the conventional bleaching process in an H_2_O_2_-containing bath (COT-H_2_O_2_(aq)). Bleaching using ozone (COT-O_3(g)_), and both ozone and hydrogen peroxide vapor (VHP) (COT-H_2_O_2(g)_-O_3(g)_), resulted only in minor changes in the surface morphology of the cellulose fibres, while for the VHP bleaching (COT-H_2_O_2(g)_), no significant changes were observed. Therefore, it may be concluded that bleaching using the gas-phase agents—ozone and VHP—is less invasive for cellulose materials than conventional bleaching. Despite the observed changes in the morphology of the surface of the fibres, none of the applied modifications caused the destruction of the fibres. Therefore, it may be concluded that the applied bleaching treatments are non-destructive.

### 3.3. Evaluation of the Whiteness and Yellowness

**Figure 4:**
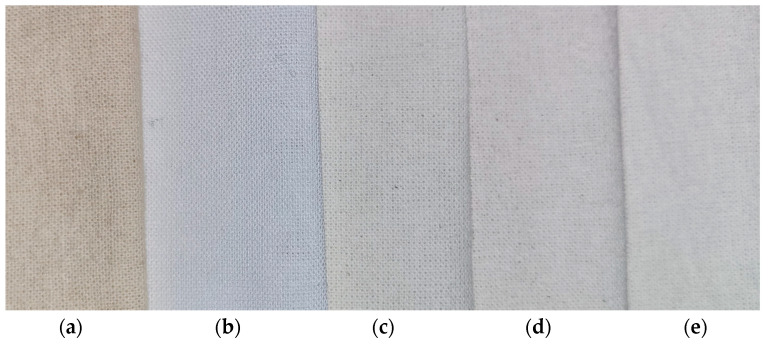
Photos of raw and bleached cotton fabric samples: (**a**) COT; (**b**) COT-H_2_O_2(as)_; (**c**) COT-H_2_O_2(g)_; (**d**) COT-O_3(g)_; (**e**) COT-H_2_O_2(g)_-O_3(g)_.

Figure 4 presents the cotton samples—raw and after bleaching process, Table 3 presents the whiteness index WI_CIE_ and yellowness index YI for the investigated cotton samples—raw and bleached.

The whiteness and yellowness indexes of the bleached cotton samples showed the significant improvement in comparison to the unmodified samples. The WI_CIE_ and YI indexes of the cotton samples bleached in comparable conditions (oxidant concentration: H_2_O_2(as)_ = 0.05 M; H_2_O_2(g)_ = 0.024 M; and O_3(g)_ = 0.21 M, temperature 35 °C and reaction time 1 h) showed the following order: WI_CIE(H2O2(as))_ > WI_CIE(O3(g))_ > WI_CIE(H2O2(g)-O3(g))_ > WI_CIE(H2O2(g)_ and YI_(H2O2(as))_ < YI_(O3(g))_ < YI _(H2O2(g)-O3(g))_ < YI _(H2O2(g)_, respectively (Table 3). The prolongation of the bleaching time for the COT-H_2_O_2(g)_ sample from 1 h to 4 h increased the WI_CIE_ values from 48.9 to 55.2 and decreased the corresponding YI values from 13.2 to 11.8 (Table 3).

The increasing ozone concentration from 0.021 Mol/L, through 0.21 Mol/L, to 0.31 Mol/L for the cotton sample bleached with ozone (COT-O_3(g)_) for 0.25 h resulted in an increase in the whiteness index from 42.2, through 50.7, to 54.6 and a decrease in the yellowness index from 13.2, through 12.4, to 11.2, respectively. Extension of the ozone bleaching time from 0.25 h to 1 h using the ozone concentration equal to 0.021 Mol/L caused a relatively small increase in the WI_CIE_ from 42.2 to 44.9 and a corresponding decrease in the YI from 13.2 to 13.1. A similar extension of the bleaching time using the ozone concentration equal to 0.21 Mol/L led to an increase in the whiteness index from 50.7 to 65.3 and a corresponding decrease in the yellowness index from 12.4 to 8.4 (Table 3).

The use of a combined bleaching process: VHP (0.024 Mol/L, 1 h) + ozone (0.021 Mol/L, 1 h) contributed to a higher value of whiteness index (WI_CIE_ = 52.8) than for each of the bleaching agents applied separately, i.e., 48.9 for VHP bleaching and 44.9 for ozone bleaching. However, for the combined bleaching process and increased ozone concentration, i.e., 0.21 Mol/L, a lower whiteness index (WI_CIE_ = 56.3) was obtained than for the bleaching using ozone alone under the same conditions (WI_CIE_ = 65.3) (Table 3).

The comparison of bleaching effectiveness of the applied procedures with the data obtained from the literature is illustrated in Table 4. 

The degree of whiteness of cotton fabric obtained after the standard bleaching process in a bath containing H_2_O_2_ depends on the quality and colour of the raw fabric as well as on the additives used in the bleaching process (Table 4). The values of the whiteness index range from 62.3 [96,97], through 64.1 [92], 65.0 [94] and 71.4–71.8 [93], up to 72.7 [this work]. The use of additional chemical compounds in the bath bleaching process, such as NaOH [95] and PAA (peracetic acid) [95], resulted in higher WI_CIE_ values of 84.1 and 72.7, respectively. In turn, the addition of MPPhA (monoperphthalic acid) resulted in a lower degree of whiteness (WI_CIE_ = 63.0) than without this addition (WI_CIE_ = 65.0) [94].

The bleaching and desizing process using glucose oxidase enzyme also resulted in a lower degree of whiteness (WI_CIE_ = 56.3) than in the case of the standard procedure (WI_CIE_ = 62.3) [96,97]. Bleaching of cotton fabric in ozonated water resulted in a whiteness index equal to 50.6, while the addition of surfactants contributed to a higher whiteness degree of the cotton fabric equal to 61.0 and even 66.5 [92] (Table 4).

Concerning the WI_CIE_ and YI factors as the primary indicators of the cotton bleaching effectiveness, we applied the corresponding conditions for the subsequent evaluation of the colour of the bleached cotton samples.

### 3.4. Evaluation of the Colour

The modification of the cellulose samples using oxidising agents in the gas phase resulted in significant changes in the CIE colour coordinates in comparison to the unmodified sample (Table 5). The bleaching processes using the oxidising agents in the gas phase resulted in the increase in the lightness (L*) of the samples from 86.00 to 88.91–93.86 for ozone, 91.53–93.08 for VHP and 92.11–92.59 for the combination of VHP and ozone. However, the highest lightness, i.e., 95.50, was achieved for the sample subjected to a conventional bleaching in the bath containing H_2_O_2_.

The values of the other CIE colour coordinates (a*, b* and chroma—C*) decreased in comparison to the unmodified sample. It means that the “less red”, “less yellow” and darker colour was obtained. On the other hand, the value of the h (hue angle) increased from 80.24 to values closer to 90, which corresponds to the yellow hue (Table 5). 

### 3.5. Determination of the Polymerisation Degree

The dependence of the polymerisation degree DP on the conditions of the applied cellulose bleaching process are summarised in Table 6. The polymerisation degree of cellulose samples after bleaching with hydrogen peroxide in both liquid and gas phases decreased significantly (by over 50%) in comparison to the unmodified sample. In the case of the conventional bleaching in the bath, the DP value was reduced from 2951 to 1234, while after VHP bleaching, it was equal to 1168. On the other hand, the treatment with ozone alone contributed to a much smaller reduction in the polymerisation degree (DP value equal to 2406). In turn, the bleaching with both oxidising agents in the gas phase, i.e., VHP and ozone, caused a drastic decrease (by 80%) in the degree of cellulose polymerisation, to the value of 579 (Table 6). This reduction in the degree of cotton polymerisation is the highest among all of the investigated procedures. It results from the fact, that according to the common knowledge, the action of strong oxidising agents (ozone and VHP) causes the chemical reconstruction of cellulose Mer cells and the formation of oxycellulose, characterized by a significantly reduced degree of polymerisation compared to the initial cellulose.

A high reduction in the DP (by 70%) was also achieved by bleaching in a bath using hydrogen peroxide with the addition of caustic soda [102], although other researchers showed a much lower reduction in the DP after a similar bleaching process [99,101,103]—by 13%, 35% and 32%, respectively, in comparison to cotton fabric before bleaching. A significant decrease in the DP (by about 52%) was noticed after the cotton bleaching in a bath with ozone in a strongly acidic environment, with an extended process time [74]. In a similar process carried out under the mild conditions, only a 25% reduction in the DP was observed [74], similar to that after the bleaching with sodium hypochlorite (NaClO) [99]. In turn, a high degree of polymerisation of cotton (over 87% in relation to the DP of the starting material) was maintained after the bleaching processes using enzymatic methods—with enzymatically (glucose oxidase; GOD) generated hydrogen peroxide [101] and/or subsequently peracetic acid [99] as well as after enzymatic desizing (α-amylase, amyloglucosidase), scoring (pectinase, glucose oxidase) and subsequent bleaching with hydrogen peroxide [103].

### 3.6. Assessment of the Mechanical Properties

The bleaching process using hydrogen peroxide, both in the gas phase and in the bath, resulted in a slight reduction in the tensile strength of the tested cotton fabric in both the longitudinal and transverse directions (Table 7). Lower values of breaking force (warp: 357 ± 7 N, weft: 376 ± 6 N) were obtained for the sample bleached with ozone. However, the greatest reduction in the tensile strength compared to the unbleached fabric—by almost 40% in the warp direction and about 20% in the weft direction—was noticed for the sample bleached using a combination of two oxidising agents in the gas phase—VHP and ozone. Such changes in the tensile strength of the cotton fabric (Table 7) correspond to changes in the value of the polymerisation degree of cellulose after various bleaching processes using oxidising agents in the gas phase (Table 6). The lower the polymerisation degree, the lower tensile strength was observed.

### 3.7. Determination of the pH of Aqueous Extract

The values of the pH of aqueous extracts of cotton samples subjected to different bleaching processes are summarised in Table 8. The pH of the aqueous extract of the unmodified woven cotton fabric was equal to 8.4. This relatively high pH value results from the use of an alkaline pre-treatment agent (Imbilan LP). After the conventional bleaching process of the cotton fabric using the bath containing H_2_O_2_, the pH of aqueous extract reached a value close to neutral, i.e., 7.4, while the VHP bleaching resulted in the slightly acidic pH, i.e., 6.7. The lowest pH value of the aqueous extract (the most acidic), equal to 5.0, was obtained for the sample bleached using ozone treatment. The pH value of the aqueous extract of cotton fabric subjected to the bleaching with both oxidising agents in the gas phase was 5.7 (Table 8). Chemical reconstruction of cellulose cells as a result of the oxidative bleaching treatment with ozone and/or VHP is associated with the ability of OH groups to oxidise and transform into aldehyde and carboxyl groups, which results in a decrease in the pH of the samples’ aqueous extracts. This was confirmed by the results presented in Table 8.

### 3.8. Measurement of the Sorption Capacity

The sorption capacity of cellulose/cotton has been the subject of many papers, in which various aspects of its application as sorbents [104,105,106], as well as models for sorption/desorption of both inorganics (water, cations) and organics (oils), were illustrated [107,108,109].

The sorption parameters of cellulose reflect a state of the surface and the morphology (an accessibility of surface hydroxyl groups, a presence of carbonyl and carboxyl functions, etc.) [110,111,112,113]. Therefore, the measurements of the sorption capacity were applied as a tool for the investigation of the surface changes of the cotton which accompanied the process of fibre bleaching. Moreover, sorption capacity is extremely important in the case of hygienic and medical applications, such as wound dressings and bandages, for which cotton is commonly applied. 

The sorption parameters of the cotton samples subjected to different bleaching processes using oxidising agents are presented in Table 9.

The highest value of maximum sorption, 38.1 µL/cm^2^, was observed for the sample subjected to a conventional bleaching process using an H_2_O_2_-containing bath. For the sample bleached using ozone or VHP and ozone combination, the maximum sorption was slightly lower, equal to 35.8 µL/cm^2^ and 36.1 µL/cm^2^, respectively. The lowest value of maximum sorption, i.e., 30.4 µL/cm^2^, was obtained for the sample subjected to VHP bleaching, and it was almost equal to the value measured for the unmodified woven cotton fabric, i.e., 30.5 µL/cm^2^. The same trend was observed for the sorption velocity, with the maximum V_30–70_ value (7.5 µL/cm^2^s) achieved for the sample subjected to conventional bleaching in bath, followed by the VHP-and-ozone-bleached sample (7.4 µL/cm^2^s), ozone-bleached sample (7.0 µL/cm^2^s) and, finally, the lowest V_30–70_ for the unmodified sample (5.5 µL/cm^2^s) and the sample after the VHP bleaching (5.3 µL/cm^2^s). At the same time, the maximum sorption velocity was the highest for the samples subjected to the bleaching using H_2_O_2_, both in bath (15.6 µL/cm^2^s) and in a gas phase (14.9 µL/cm^2^s). In turn, the lowest maximum sorption velocity was measured for the sample modified using ozone (10.1 µL/cm^2^s) and a combination of both oxidising agents, i.e., VHP and ozone (9.7 µL/cm^2^s). The total sorption time decreased for all of the bleached cotton samples in comparison to the unmodified one (23.5 s). The lowest sorption time was obtained for the sample treated using ozone (11.6 s) and using a combination of VHP and ozone (11.4 s), while for the samples bleached using H_2_O_2_, either in bath or gas phase, it was similar and equal to 16.0 s and 17.5 s, respectively.

### 3.9. Microbiological Assessment of the Decontamination Efficiency

Ozone and hydrogen peroxide are extensively used antimicrobial chemicals for preservative, disinfection and sterilization applications [114]. They exhibit the broad-spectrum antimicrobial activity, illustrated by over 2000 documents for hydrogen peroxide [115] and over 200 documents for ozone [116] abstracted in the Scopus base, respectively. Both oxidants also exhibit antiviral potential [117,118]. There were also papers on the antibacterial cotton fabric prepared with other antibacterial agents [119,120,121,122]

The results of the microbiological tests (Table 10) indicated that the gas-phase bleaching process of cellulose samples using oxidising agents like ozone and vaporised hydrogen peroxide resulted in high biocidal efficiency against Gram (+) *S. aureus* (ATCC 6538) and Gram (-) *E. coli* (ATCC 11229) bacteria strains. The use of VHP was also applied earlier in the process of waste-free, low-temperature bleaching with simultaneous disinfection [Gram (+) and Gram (-) bacteria, moulds, fungi and spore forms] of cellulose fibre materials [47]. Due to the fact that after the bleaching using each bleaching agent—VHP and ozone separately—a very good biocidal effect was achieved against the tested microorganisms, and antimicrobial activity tests were no longer performed after the combined bleaching process using both—VHP and ozone. It can be concluded that the treatment of cotton using gaseous ozone and VHP provides a simultaneous bleaching and disinfection effect.

## 4. Conclusions

In this paper, the set of eco-friendly cotton bleaching procedures based on the use of gaseous hydrogen peroxide and ozone (namely COT-H_2_O_2(g),_ COT-O_3(g)_ and COT-H_2_O_2(g)_-O_3(g)_) was presented.

These procedures were carried out at nearly ambient temperature (35 °C), close to neutral pH, over a relatively short reaction time (0.25–4 h), using low total water consumption (mainly limited to the preliminary/final washings).

The significant improvement in the whiteness index (WI_CIE_) and the reduction in the yellowness index (YI) of the bleached cotton samples in comparison to the unmodified samples was shown.

Scanning Electron Microscopy of the cotton fibres showed minor changes in the fibre morphology, suggesting that the presented bleaching procedures are non-invasive.

The polymerisation degree of the cotton samples decreased substantially after the bleaching (over 50% for both COT-H_2_O_2(as)_ and COT-H_2_O_2(g)_; 21% for COT-O_3(g)_ and 80% for COT-H_2_O_2(g)_-O_3(g)_) due to the applied oxidation treatment. These changes were also accompanied by the tensile strength decrease of the tested cotton samples. The reduction in the tensile strength corresponded to the decrease in the polymerisation degree, which is turn was associated with the chemical reconstruction of cellulose Mer cells and the formation of oxycellulose.

The sorption parameters of the samples COT-H_2_O_2(g)_, COT-O_3(g)_ and COT-H_2_O_2(g)_-O_3(g)_ improved—the amount of adsorbed moisture (maximum sorption) and the sorption velocity increased, and the sorption time was shortened. This is of the utmost importance for medical and hygienic applications of cotton.

The results of microbiological tests showed high decontamination efficiency of gas-phase bleaching procedures (COT-H_2_O_2(g)_, COT-O_3(g)_ and COT-H_2_O_2(g)_-O_3(g)_) against *S. aureus* and *E. coli*.

Comparison of the abovementioned findings leads to the conclusion that the bleaching procedures using gaseous oxidants (COT-H_2_O_2(g)_, COT-O_3(g)_ and COT-H_2_O_2(g)_-O_3(g)_) are non-destructive and allow to obtain good functional properties, comparable with those reached for the standard aqueous hydrogen peroxide bleaching (COT-H_2_O_2(as)_). These procedures, due to lower bleaching temperature (35 °C vs. 98 °C) and minimal water consumption, have economic and environmental advantages, which advocates for their use in semi-industrial applications. Moreover, it has been shown that the treatment of cotton fabrics with the gaseous oxidants (COT-H_2_O_2(g)_, COT-O_3(g)_ and COT-H_2_O_2(g)_-O_3(g)_) allows to obtain a simultaneous bleaching and disinfection effect combined with superior whiteness and sorption capacity. 

However, the achieved polymerisation degree and tensile strength are lowered. Therefore, the application of the applied procedures is recommended for the production of textile materials for medical and hygienic products where medical properties (antimicrobial resistance, high whiteness and good sorption) are more important than mechanotechnical properties.

## Figures and Tables

**Table 1:** The main coloured constituents of cotton fibres.

The Main Coloured Constituents of Cotton Fibres				
Comp.	Cellulose	Lignin	Flavonoids	Cotton fibre wax
	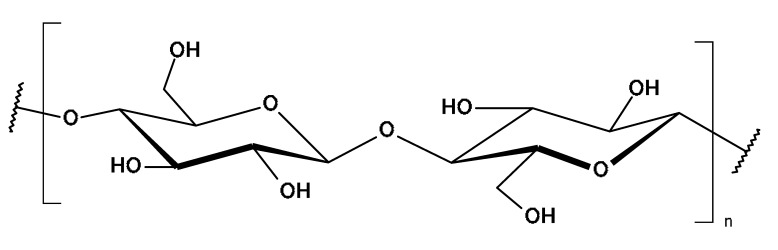	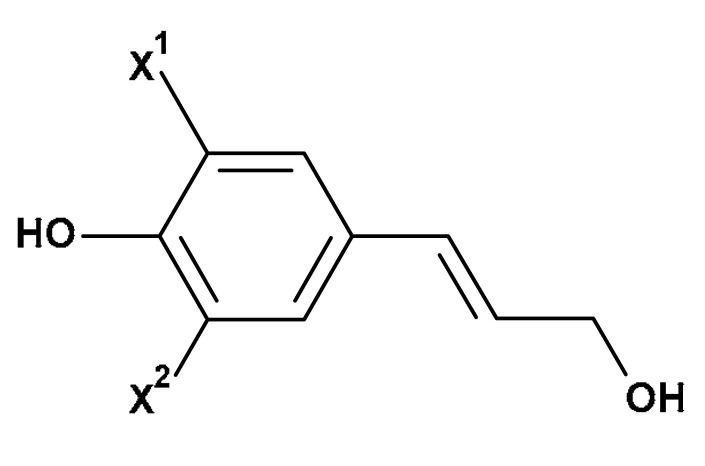	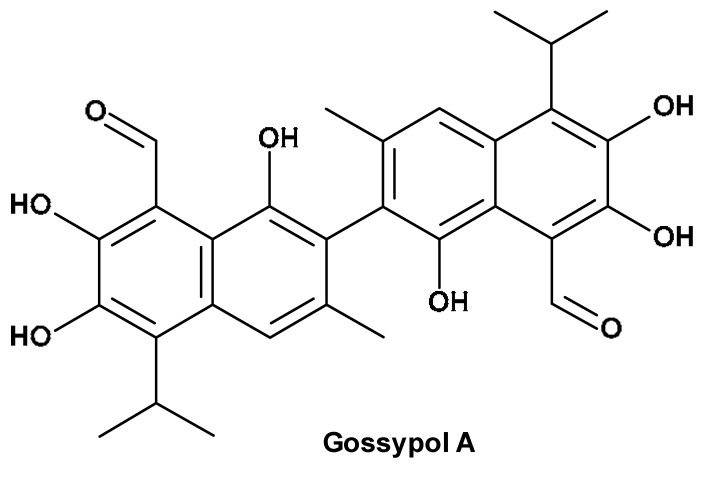	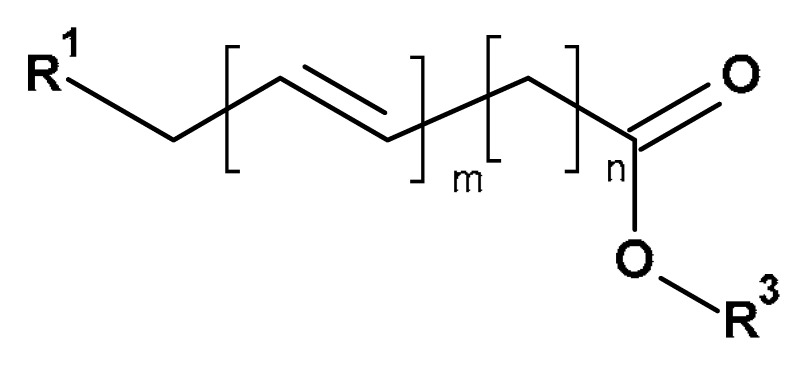
Con. [%]	83–90 [12,13]	0.8 [12,14]	0.5–8 [15,16,17,18]	0.4–1.0 [12,19,20]

Comp.—component; Con.—content.

**Table 2:** Cotton bleached samples and conditions of bleaching procedures applied.

No.	Sample Abbreviation	Bleaching							
		Oxidants						Conditions	
		H_2_O_2(as)_		H_2_O_2(g)_		O_3(g)_		Temp. [°C]	Time [h]
		ppm	Mol/L	ppm	Mol/L	ppm	Mol/L		
1.	COT	-		-		-		-	-
2.	COT-H_2_O_2(as)_ ^(1)^	1680	0.05					98	1
3.	COT-H_2_O_2(g)_			800	0.024			35	1–4
4.	COT-O_3(g)_					1000–15,000	0.021–0.031	35	0.25–1
5.	COT-H_2_O_2(g)_-O_3(g)_			800	0.024	1000–10,000	0.021–0.21	35	1

Abbreviations: COT—Unbleached 100% plain woven cotton fabric; COT-H_2_O_2(as)_—Plain woven cotton fabric (100%) after conventional bleaching in H_2_O_2_ bath; ^(1)^ liquor ratio COT: bath = 1:10; a bath contained 4 g/L of 30% H_2_O_2(as)_; COT-H_2_O_2(g)_—Plain woven cotton fabric (100%) after VHP bleaching (35 °C, 1 h or 4 h); COT-O_3(g)_—Plain woven cotton fabric (100%) after ozone bleaching (35 °C, 0.25 h or 1 h). COT-H_2_O_2(g)_-O_3_—Plain woven cotton fabric (100%) after sequential VHP bleaching (35 °C, 1 h) and ozone bleaching (35 °C, 1 h).

**Table 3:** Whiteness index (WI_CIE_) and yellowness index (YI) for cotton fabric—raw and bleached samples.

No.	Sample Abbreviation	Bleaching Conditions					Determined Data	
		Oxidant [Mol/L]			Conditions			
		H_2_O_2(as)_	H_2_O_2(g)_	O_3(g)_	Temp. [°C]	Time [h]	WI_CIE_	YI
1	COT				-	-	24.1	19.1
2	COT-H_2_O_2(as)_ ^(1)^	0.05			98	1	72.7	6.8
3.1	COT-H_2_O_2(g)_		0.024		35	1	48.9	13.2
3.2			0.024		35	4	55.2	11.8
4.1	COT-O_3(g)_			0.021	35	0.25	42.2	13.2
4.2				0.021	35	1	44.9	13.1
4.3				0.21	35	0.25	50.7	12.4
4.4				0.21	35	1	65.3	8.4
4.5				0.31	35	0.25	54.6	11.2
5.1	COT-H_2_O_2(g)_-O_3(g)_		0.024		35	1		
				0.021	35	1	52.8	12.1
5.2			0.024		35	1		
				0.21	35	1	56.3	10.8

Abbreviations: COT—Unbleached 100% plain woven cotton fabric; COT-H_2_O_2(as)_—Plain woven cotton fabric (100%) after conventional bleaching in H_2_O_2_ bath; ^(1)^ liquor ratio COT: bath = 1:10; a bath contained 4 g/L (0.05 Mol/L) of 30% H_2_O_2(as)_; COT-H_2_O_2(g)_—Plain woven cotton fabric (100%) after VHP bleaching (35 °C, 1 h or 4 h); COT-O_3(g)_—Plain woven cotton fabric (100%) after ozone bleaching (35 °C, 0.25 h or 1 h). COT-H_2_O_2(g)_-O_3_—Plain woven cotton fabric (100%) after sequential VHP bleaching (35 °C, 1 h) and ozone bleaching (35 °C, 1 h). WI_CIE_—whiteness index; YI—yellowness index (E313 Yellowness Index).

**Table 4:** Comparison of whiteness index WI_CIE_ for cotton samples after representative bleaching processes using different oxidising agents.

Sample Abbreviation ^(a,b)^	WI_CIE_ ^(c)^					
	This Work	Literature Data				
		[92] ^(1,2)^	[93] ^(3)^	[94] ^(4,5)^	[95] ^(6–8)^	[96,97] ^(9,10)^
COT	**24.** **1**	2.1	1.2		11.1	
COT-NaOH					19.5 ^(6)^	
COT-NaOH-H_2_O_2(as)_					84.1 ^(7)^	
COT-NaOH-PAA_(as)_					72.7 ^(8)^	
COT-H_2_O_2(as)_-MPPhA				63.0 ^(4)^		
COT-H_2_O_2(as)_	**72.** **7**	64.1	71.4–71.8	65.0 ^(5)^		62.3 ^(9)^
COT-H_2_O_2(as)_-GOD						56.3 ^(10)^
COT-H_2_O_2(g)_	**48.9–55.2**					
COT-O_3(g)_	**42.2–65.3**					
COT-H_2_O-O_3(g)_		50.6 ^(1)^; 56.1–66.5 ^(2)^				
COT-H_2_O_2(g)_-O_3(g)_	**52.8–56.3**					

^(a)^ Experimental conditions used: ^(1)^ Ozone dose, 10 g/h; 0.75 h, temp.: 30 °C, liquor ratio 1:10; ^(2)^ With addition of 0.01 g/L of surfactant (anionic, cationic and/or non-ionic); ^(3)^ Bleaching solution: activator TBCC (8 g/L), chelate DTPMP (29 g/L), H_2_O_2_ (100 g/L). Conditions: pH 11.7, 8 h; ^(4)^ Bleaching solution: NaOH (2 g/L); H_2_O_2_ (1.5 g/L); PhA (4 g/L). Conditions: 70 °C, 1–1.25 h; ^(5)^ Bleaching solution: NaOH (4 g/L); H_2_O_2_ (2.7 g/L). Conditions: 70 °C, 1–1.25 h; ^(6)^ Scouring solution: NaOH (3 g/L). Conditions: pH 12.5; 95 °C, 0.7 h; ^(7)^ Bleaching solution: NaOH (4 g/L); H_2_O_2_ (2.7 g/L); EDTA (0.2 g/L). Conditions: pH 12.5; 95 °C, 0.7 h; ^(8)^ Bleaching solution: NaOH (2.2 g/L); PAA (2.3 g/L); Conditions: pH 8; 55 °C, 0.7 h; ^(9)^ Bleaching solution: NaOH (4% o.w.f.); NaOH (4.2 g/L). Conditions: pH 8; 90–100 °C, 0.5 h; ^(10)^ Bleaching and desizing procedure: COT was treated with Dextrozyme DX (1% o.w.f), 65 °C, 0.5 h; followed by bleaching by GOD addition, conditions 100 °C; ^(b)^Abbreviations: Dextrozyme DX—myloglucosidase/pullanase enzyme; GOD—glucose oxidase enzyme; MPPhA—monoperphthalic acid; PAA—peracetic acid; PhA—phthalic anhydride; TAED—tetraacetylethylenediamine; TBCC N-[4-(triethylammoniomethyl) benzyl]caprolactam chloride; DTPMP—(diethylenetriamine)pentakis (methylphosphonic acid); ^(c)^ WI_CIE_ literature data were approximated to the first decimal place.

**Table 5:** CIE colour coordinates for cotton samples subjected to different bleaching processes using oxidising agents.

Sample	CIE Colour Coordinates										
	CIELAB and CIELCH Values					Colour Difference					
	L*	a*	b*	C*	h	DE*	DL*	Da*	Db*	DC*	Dh
**COT**	86.00	1.54	8.92	9.05	80.24	-	-	-	-	-	-
**COT-H_2_O_2(as)_**	95.50	0.06	3.56	3.56	89.09	11.0	9.49	−1.48	−5.36	−5.49	0.88
**COT-H_2_O_2(g)_**—1 h	91.53	0.67	6.57	6.61	84.15	6.07	5.53	−0.86	−2.35	−2.45	0.53
**COT-H_2_O_2(g)_**—4 h	93.08	0.32	6.05	6.06	87.01	7.73	7.27	−1.22	−2.87	−2.99	0.88
**COT-O_3(g)_**—0.021 Mol/L; 0.25 h	88.91	1.16	6.64	6.74	80.09	3.72	2.91	−0.37	−2.28	−2.31	−0.02
**COT-O_3(g)_**—0.021 Mol/L; 1 h	89.40	1.18	6.33	6.43	79.47	4.29	3.40	−0.36	−2.60	−2.62	−0.10
**COT-O_3(g)_**—0.21 Mol/L; 0.25 h	91.14	0.93	5.99	6.06	81.18	5.95	5.14	−0.61	−2.93	−2.99	0.12
**COT-O_3(g)_**—0.021 Mol/L; 1 h	93.86	0.34	4.24	4.26	85.37	9.22	7.86	−1.19	−4.68	−4.80	0.56
**COT-H_2_O_2(g)_-O_3(g)_**—0.021 Mol/L O_3_	92.11	0.57	6.06	6.09	84.61	6.81	6.11	−0.96	−2.86	−2.97	0.57
**COT-H_2_O_2(g)_-O_3(g)_**—0.21 Mol/L O_3_	92.59	0.65	5.56	5.60	83.38	7.44	6.58	−0.89	−3.36	−3.45	0.39

Colour Parameters: L*—lightness; a*—green/red; b*—blue/yellow; C*—chroma; h—hue angle; DE*—total colour difference; DL*—difference in lightness; Da*—difference in green./red parameters; Db*—difference in green/yellow parameters; DC*—difference in chroma; Dh—difference in hue angles. **COT-H_2_O_2(as)_** (H_2_O_2_: 0.05 Mol/L; 98 °C; 1 h); **COT-H_2_O_2(g)_** (H_2_O_2_: 0.024 Mol/L; 35 °C; 1 h and 4 h); **COT-O_3(g)_** (O_3_: 0.021 or 0.21 Mol/L; 35 °C; 0.25 h or 1 h). **COT-H_2_O_2(g)_-O_3(g_**_)_ (H_2_O_2_: 0.024 Mol/L; 35 °C; 1 h; O_3_: 0.021 or 0.21 Mol/L; 35 °C; 1 h).

**Table 6:** Changes in the polymerisation degree of cotton samples subjected to different bleaching processes using representative oxidising agents.

Cotton Treatment	Cotton Treated Polymerisation Degree [DP_bc_/(DP_ubc_)] ^(1)^							
	This Work	Literature Data						
		[74] ^(2,3)^	[98] ^(4)^	[99] ^(5)^	[100] ^(6)^	[101] ^(7)^	[102] ^(8,9)^	[103] ^(10,11)^
COT-H_2_O_2(as)_	**1234/** **(2951)**	2676/ (3190)	1856/ (2176) ^(2)^; 1679/ (2176) ^(4)^			1946/(2232)		1593/ (2312)
COT-H_2_O_2_-NaOH				2155/ (2470)		1445/ (2232)	1180/ (3850) ^(8)^	1593/ (2312) ^(11)^
COT-H_2_O_2(g)_	**1168/** **(2951)**							
COT-O_3(g)_	**2406/** **(2951)**							
COT-H_2_O_2(g)_—O_3(g)_	**579/** **(2951)**							
COT-H_2_O-O_3(g)_		2419/ (3190) ^(1)^; 1673/ (3190) ^(2)^						
COT-PAA-H_2_O_2_					2526–2463/ (2845)	2047/ (2232)		
COT-NaClO-H_2_O_2_				1860/ (2470)				
COT-H_2_O_2_-ENZ						1946–2221/ (2232)	3750/ (3850) ^(9)^	2038–2187/ (2312)

^(1)^ Polymerisation degree of bleached cotton (DP_bc_) vs. polymerisation degree of unbleached cotton (DP_ubc_) [DP_bc_/(DP_ubc_)]; ^(2,3)^ Ozone treatment: 45 min treatment time at pH 5 at 25–30 °C with doses: ^(1)^ 2 g/h; ^(2)^ 10 g/h; ^(4)^ Cotton pre-treated with alkali (0.2%) at pH 11 and treated with H_2_O_2_ doses of 2% and 4% (on fibre); ^(5)^ Cotton bleaching at 20 °C; 35 min; ^(6)^ Cotton bleaching with PAA—2.25 g/L; pH 7, 40 min, at 40 °C and 80 °C; ^(7–9)^ Cotton bleaching with enzymatically generated H_2_O_2_; ^(7)^ Cotton enzymatic desizing (mixtures of α-amylases, amyloglucosidases and pullanases) and scouring with pectinases (50 °C, pH 5; 1 h). Bleaching with enzymatically (glucose oxidase; GOD) generated hydrogen peroxide and/or subsequently peracetic acid (50 °C, pH 5–7.5, 1 h); ^(8,9)^ Cotton bleaching with enzymatically generated H_2_O_2_: ^(6)^ 80–95 °C; LR = 0.05; ^(7)^ ENZ—glucose oxidase, pH 7, at 85–90 °C for 1–2 h; ^(10,11)^ Cotton enzymatic desizing (α-amylase, amyloglucosidase) and scoring (pectinase, glucose oxidase). Bleaching with hydrogen peroxide; ^(11)^ Conventional process: consecutive enzymatic desizing with α-amylase, alkaline scouring, and bleaching with hydrogen peroxide (80–95 °C, pH 11, 1 h); **This work applied conditions: COT-H_2_O_2(as)_** (H_2_O_2_: 0.05 Mol/L; 98 °C; 1 h); **COT-H_2_O_2(g)_** (H_2_O_2_: 0.024 Mol/L; 35 °C; 4 h); **COT-O_3(g)_** (O_3_: 0.21 Mol/L; 35 °C; 1 h); **COT-H_2_O_2(g)_-O_3(g)_** (H_2_O_2_: 0.024 Mol/L; 35 °C; 1 h; O_3_: 0.21 Mol/L; 35 °C; 1 h).

**Table 7:** Maximum force and relative elongation at maximum force for cotton samples after different bleaching processes using oxidising agents.

Cotton Sample	Maximum Force [N]		Rel. Elong. _(max. force)_ [%]	
	Warp	Weft	Warp	Weft
**COT**	510 ± 10	410 ± 9	10.5 ± 0.9	24.0 ± 1.2
**COT-H_2_O_2 (as)_**	430 ± 8	380 ± 8	15.5 ± 1.4	25.5 ± 1.1
**COT-H_2_O_2(g)_**	470 ± 7	390 ± 8	10.5 ± 1.0	25.0 ± 1.6
**COT-O_3(g)_**	357 ± 7	376 ± 6	14.8 ± 1.3	25.7 ± 1.5
**COT-H_2_O_2(g)_-O_3(g)_**	321 ± 6	332 ± 7	13.1 ± 1.2	15.6 ± 1.3

Warp—longitudinal direction; Weft—transverse direction; This work applied conditions: **COT-H_2_O_2(as)_** (H_2_O_2_: 0.05 Mol/L; 98 °C; 1 h); **COT-H_2_O_2(g)_** (H_2_O_2_: 0.024 Mol/L; 35 °C; 4 h); **COT-O_3(g)_** (O_3_: 0.21 M; 35 °C; 1 h); **COT-H_2_O_2(g)_-O_3(g)_** (H_2_O_2_: 0.024 Mol/L; 35 °C; 1 h; O_3_: 0.21 Mol/L; 35 °C; 1 h).

**Table 8:** Values of the pH of aqueous extracts of cotton samples subjected to different bleaching processes.

Sample	pH of Aqueous Extract
COT	8.4
COT-H_2_O_2(as)_	7.4
COT-H_2_O_2(g)_	6.7
COT-O_3(g)_	5.0
COT-H_2_O_2(g)_-O_3(g)_	5.7
H_2_O_2/(0.3%; 0.1M)_	6.4

This work applied conditions: **COT-H_2_O_2(as)_** (H_2_O_2_: 0.05 Mol/L; 98 °C; 1 h); **COT-H_2_O_2(g)_** (H_2_O_2_: 0.024 Mol/L; 35 °C; 4 h); **COT-O_3(g)_** (O_3_: 0.21 Mol/L; 35 °C; 0.5 h); COT-H_2_O_2(g)_-O_3(g)_ (H_2_O_2_: 0.024 Mol/L; 35 °C; 1 h; O_3_: 0.21 Mol/L; 35 °C; 0.5 h).

**Table 9:** Sorption parameters of cotton samples subjected to different bleaching processes using oxidising agents.

Sample	Sorption Parameters			
Sample Abbreviation	S_max_ [µL/cm^2^]	V_max_ [µL/cm^2^s]	V_30–70_ [µL/cm^2^s]	t_max_ [s]
COT	30.5	11.8	5.5	23.5
COT-H_2_O_2(as)_	38.1	15.6	7.5	16.0
COT-H_2_O_2(g)_	30.4	14.9	5.3	17.5
COT-O_3(g)_	35.8	10.1	7.0	11.6
COT-H_2_O_2(g)_-O_3(g)_	36.1	9.7	7.4	11.4

Sorption parameters: S_max_—Maximum sorption; V_max_—Maximum sorption velocity; V_30–70_—Sorption velocity; t_max_—Total sorption time. This work applied conditions: **COT-H_2_O_2(as)_** (H_2_O_2_: 0.05 Mol/L; 98 °C; 1 h); **COT-H_2_O_2(g)_** (H_2_O_2_: 0.024 Mol/L; 35 °C; 4 h); **COT-O_3(g)_** (O_3_: 0.21 Mol/L; 35 °C; 0.5 h); **COT-H_2_O_2(g)_-O_3(g)_** (H_2_O_2_: 0.024 Mol/L; 35 °C; 1 h; O_3_: 0.21 Mol/L; 35 °C; 1 h).

**Table 10:** Efficiency of decontamination of cotton samples during different bleaching processes using oxidising agents.

Cotton Bleaching/Disinfection ^a,b^				Disinfection Test	
Sample	Oxidant Concentration		Time [h]	*E. coli*	*S. aureus*
	ppm	Molar			
COT ^a^	-		-	Growth: Turbidity, sediment	Growth: Turbidity, sediment
COT-H_2_O_2(as)_ ^b^	1680	0.05	1	No growth: Clear medium	No growth: Clear medium
COT-H_2_O_2(g)_ ^b^	800	0.024	0.33		
COT-O_3(g)_^b^	10,000	0.21	0.33		

^a^ Control sample. ^b^ Decontaminated sample (after bleaching/disinfection).

## Data Availability

Data are contained within the article.
